# Mechanical ventilation in patients with acute ischaemic stroke: from pathophysiology to clinical practice

**DOI:** 10.1186/s13054-019-2662-8

**Published:** 2019-12-02

**Authors:** Chiara Robba, Giulia Bonatti, Denise Battaglini, Patricia R. M. Rocco, Paolo Pelosi

**Affiliations:** 1 Anesthesia and Intensive Care, San Martino Policlinico Hospital, IRCCS for Oncology and Neurosciences, University of Genoa, Largo Rosanna Benzi, 15, 16100 Genoa, Italy; 2 Department of Surgical Sciences and Integrated Diagnostics, University of Genoa, Genoa, Italy; 3 Laboratory of Pulmonary Investigation, Carlos Chagas Filho Institute of Biophysics, Federal University of Rio de Janeiro, Rio de Janeiro, Brazil

**Keywords:** Stroke, Brain injury, Mechanical ventilation, Brain-lung crosstalk, Intensive care unit

## Abstract

Most patients with ischaemic stroke are managed on the ward or in specialty stroke units, but a significant number requires higher-acuity care and, consequently, admission to the intensive care unit. Mechanical ventilation is frequently performed in these patients due to swallowing dysfunction and airway or respiratory system compromise. Experimental studies have focused on stroke-induced immunosuppression and brain-lung crosstalk, leading to increased pulmonary damage and inflammation, as well as reduced alveolar macrophage phagocytic capability, which may increase the risk of infection. Pulmonary complications, such as respiratory failure, pneumonia, pleural effusions, acute respiratory distress syndrome, lung oedema, and pulmonary embolism from venous thromboembolism, are common and found to be among the major causes of death in this group of patients. Furthermore, over the past two decades, tracheostomy use has increased among stroke patients, who can have unique indications for this procedure—depending on the location and type of stroke—when compared to the general population. However, the optimal mechanical ventilator strategy remains unclear in this population. Although a high tidal volume (*V*_T_) strategy has been used for many years, the latest evidence suggests that a protective ventilatory strategy (*V*_T_ = 6–8 mL/kg predicted body weight, positive end-expiratory pressure and rescue recruitment manoeuvres) may also have a role in brain-damaged patients, including those with stroke. The aim of this narrative review is to explore the pathophysiology of brain-lung interactions after acute ischaemic stroke and the management of mechanical ventilation in these patients.

## Background

Acute ischaemic stroke (AIS) is one of the major causes of morbidity and mortality worldwide [1] and one of the leading causes for admission to neurological intensive care units (NICUs) [2]. Over the last decades, the incidence of stroke has been increasing, and despite an overall decrease in mortality, it is still the leading cause of severe disability in the adult population [1]. Approximately 80% of all strokes are ischaemic; other major types include intracerebral (ICH) and intraventricular (IVH) haemorrhage, cerebral venous and sinus thrombosis, and subarachnoid haemorrhage (SAH) secondary to aneurysm leak or rupture [3]. The location of the stroke is probably the most relevant factor related to the need for mechanical ventilation (MV), rather than the particular type of cerebrovascular pathology. In this context, impairment of the brain areas that regulate the level of consciousness (thalami, the limbic system, the reticular formation in the brainstem), breathing (respiratory centres in the cortex, pons, and medulla), and swallowing (medulla and brainstem connections) increases the risk of respiratory failure [4].

Pulmonary complications—such as respiratory failure, pneumonia, pleural effusion, acute respiratory distress syndrome (ARDS), pulmonary oedema, and pulmonary embolism from venous thromboembolism—may occur in this group of patients and are associated with a high risk of mortality [5, 6]. In particular, stroke-associated pneumonia (SAP) is described as an independent risk factor for unfavourable outcome and death [7, 8]. Since the most frequent extra-cerebral complication of neurological ICU patients is respiratory failure [5], the development of new mechanical ventilation strategies may potentially improve their outcome [4]. So far, few studies have addressed the best respiratory management of patients with AIS. The aim of this manuscript is to review and describe the pathophysiology underlying the development of pulmonary complications and respiratory failure after AIS and the different ventilator strategies in this population, focusing on the risk related to oxygen (O_2_) therapy and the application of protective lung ventilation. For this purpose, we performed a literature search of four electronic databases (PubMED, Scopus, ScienceDirect, Web of Science), using the following terms: “mechanical ventilation” and “stroke”. Titles and abstracts were retrieved and independently assessed for eligibility by two authors (CR, GB). Disagreements were resolved by discussion and consensus agreement and, if required, input from a third author (DB).

This narrative review focuses on the information specific to stroke patients that do not have brain trauma or intracranial pressure issues, including the concern over the need for tracheostomy as an intervention to prevent aspiration pneumonia rather than for primarily pulmonary issues. We also aimed to highlight the different needs of specific subsets of stroke victims that distinct profiles of neurological impairment—such as decreased level of consciousness, cranial nerve deficits, and central swallow concerns—in order to provide a good roadmap to guide the generalist intensivist.

## The pathophysiology of stroke and the risk of pulmonary complications

### Brain-lung crosstalk

The concept of brain-organ crosstalk has been widely investigated [9, 10]. Severe brain-damaged patients are commonly unable to protect their airway and therefore are often admitted to the NICU for mechanical ventilation. Prolonged MV is associated with increased risk of developing ventilator-associated lung injury, ARDS, pneumonia, and neurogenic pulmonary oedema [11]. Moreover, ARDS has detrimental effects on the brain, acting synergistically with intracranial hypertension to cause hippocampal damage. A study on rats revealed that focal ischaemic stroke alters the respiratory pattern, induces oedema and inflammation, and decreases the phagocytic capability of alveolar macrophages [6]. Remarkably, all of these changes occurred within 24 h after the induction of cerebral ischaemia, which is in agreement with the rapid onset of respiratory failure in stroke patients [12].

The mechanism of lung damage after brain injury is described through a “double-hit model”: the catecholamine storm and the systemic production of inflammatory mediators (first hit) create a systemic inflammatory environment which increases pulmonary vascular hydrostatic pressure and activates biological mechanisms that make the lung more susceptible to mechanical and non-mechanical insults (second hit), including mechanical ventilation [13]. On the other hand, lung injury can damage the brain through a complex interaction between the autonomic, neuro-inflammatory, neuroendocrine, and immunologic pathways [14]. Fries et al. [15] compared pigs with hypoxaemia induced by a lavage model of acute lung injury versus pigs with the same degree of hypoxaemia induced by a reduction in the fraction of inspired oxygen (FiO_2_). Brain damage was greater in the first group, suggesting that acute lung injury per se leads to neuro-pathologic changes regardless of the occurrence of hypoxaemia. The lungs can communicate with the brain through the autonomic and sympathetic nervous system, leading to a neuro-inflammatory process which triggers the activation of the cytokine and complement cascades [16], thus contributing to the development of brain oedema and apoptotic cell death.

### Immunological response after stroke

**Fig. 1:**
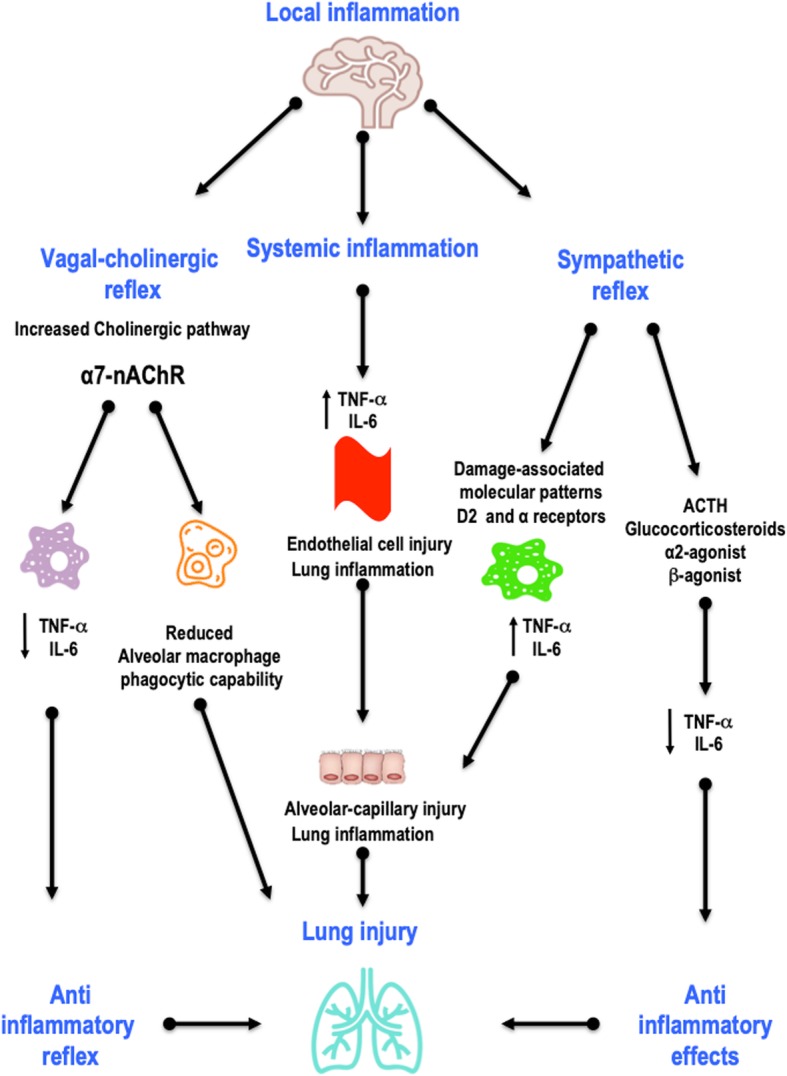
Brain-systemic crosstalk. After stroke, stimulation of the vagus nerve through nicotinic acetylcholine receptor α7 (nAChRα7) induces microglial activation, causing reduced alveolar macrophage phagocytic capability and reducing circulating levels of interleukin IL-6 and tumour necrosis factor TNF-α, thus leading to an anti-inflammatory reflex and lung injury. On the other hand, systemic inflammation consequent to stroke leads to an increased release of inflammatory mediators such as IL-6 and TNF-α, resulting in lung inflammation and alveolar-capillary injury. Finally, a sympathetic response with increased expression of inflammatory mediators and hypothalamic-pituitary-adrenal axis activation induces elevated glucocorticoid secretion, which might be associated with secondary infections and poor outcome

**Fig. 2:**
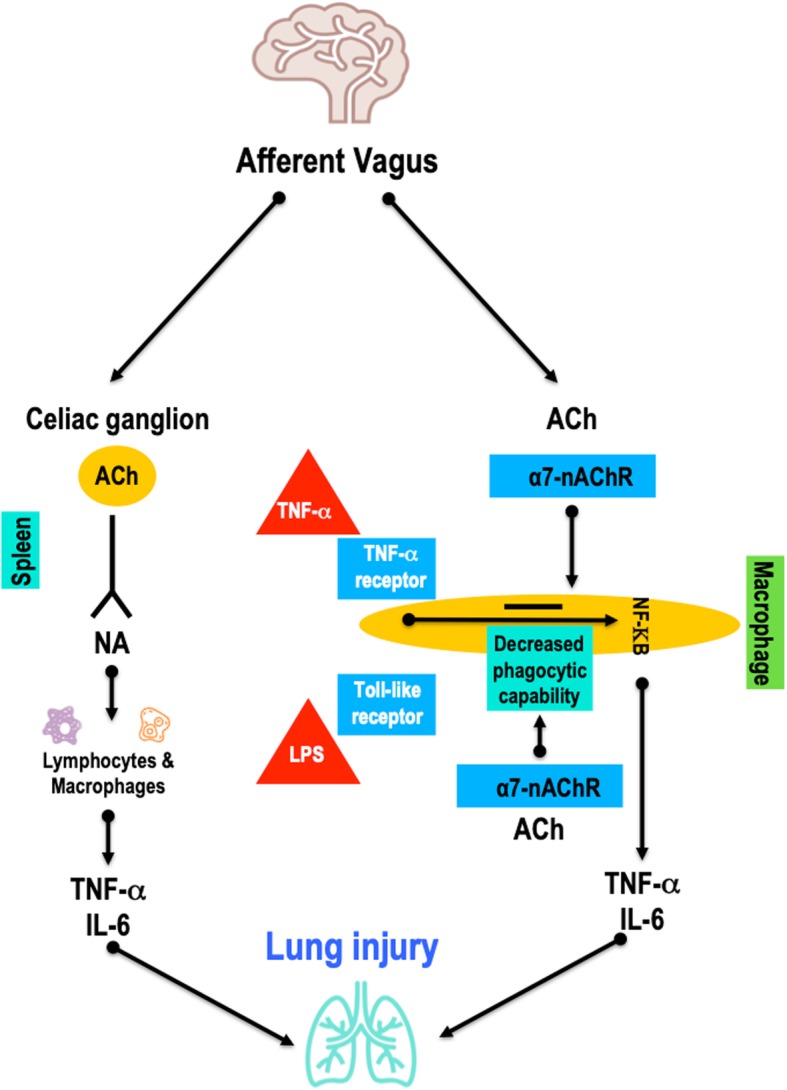
The role of the vagal pathways in the development of lung injury. The healthy brain can control excess cytokine production via an inflammatory reflex of the vagus nerve (by activation of the afferent vagus through the celiac ganglion). Stimulation of the vagus nerve through nicotinic acetylcholine receptor α7 (nAChRα7) regulates microglial activation in the brain, protects neuronal cells from oxidative stress, and improves functional recovery, contributing to immunosuppression. Expression of nAChRα7 on alveolar macrophages and epithelial cells induces a reduction of inflammation in the lungs, and, by suppressing the production of pro-inflammatory cytokines (IL-6, TNF-α) through lipopolysaccharides (LPS) and *nuclear factor* kappa-light-chain-enhancer of activated B cells (NFK-B), impairs host defence during inflammatory conditions. Altogether, vagal stimulation during stroke blunts macrophage capabilities, with increased risk of infection and lung injury, while paradoxically inducing a higher anti-inflammatory response and thus decreasing the risk of lung injury. The balance between these two pathways accounts for the occurrence, or not, of lung injury. Ach, acetylcholine; NA, noradrenaline

AIS produces profound local and systemic immune responses that engage all major innate and adaptive immune compartments. After stroke, damage to the blood-brain barrier (BBB) leads to recruitment of resident (astrocytes and microglia) and peripheral (primarily neutrophils and monocytes) immune cells to the affected area [17]. The result is a reduction in circulating immune cells and a depression of peripheral immunity that increases the susceptibility to infections [8]. A large body of experimental and clinical evidence supports the hypothesis of a downregulation of systemic cellular immune responses, with a rapid numerical decrease in peripheral blood lymphocyte subpopulations and functional deactivation of monocytes [18]. These changes are most prominent among patients with larger strokes and are more likely after strokes located in the insular cortex [8]. Experimental and clinical studies in AIS have also reported increased levels of circulating catecholamines and their correlation with lymphopaenia. However, the mechanisms of humoral immunosuppression and bacterial infections remain controversial [10, 19]. In post-stroke mice, the use of β-adrenergic receptor antagonists reduces bacterial complications and mortality rates, which may suggest the importance of the catecholamine pathway and vagal response in the immunosuppression process [19] (Figs. 1 and 2).

### Stroke-associated pneumonia

Aspiration pneumonia is one of the major causes of respiratory failure after stroke and can be related to a decreased level of consciousness, with impaired swallowing and dysphagia [8]. However, the incidence of pneumonia in stroke patients is higher when compared to other groups of patients who suffer from dysphagia or compromised level of consciousness, thus suggesting that other mechanisms are involved, such as immune activation and alteration in lung injury pathogenesis [20]. PREDICT, a prospective observational multicentre study, confirmed that both dysphagia and stroke-induced immunodepression syndrome are independent risk factors for aspiration pneumonia [20]. The incidence of SAP ranges between 3.9 and 56.6%, with a higher incidence in the ICU compared to stroke units or wards [8]. Several studies have developed clinical scores to predict SAP with the aim of improving risk stratification in patients with acute stroke [21, 22], but their use in the clinical setting is uncommon. In current practice, biomarkers such as C-reactive protein, stroke severity, dysphagia, and the Centres for Disease Control and Prevention criteria [22] are more frequently used. The most widely recognised measure when acute stroke patients are admitted is nil by mouth status until a swallowing assessment can be completed [8]. Early mobilisation is recommended as it decreases the risk of SAP, while the benefit of antibiotic use in the acute phase remains unproven [23].

### Stroke-associated dysphagia

Prediction of extubation success and safe extubation are important in stroke patients. A prospective observational study [24] that focused on extubation readiness in stroke patients showed that patients who failed in swallowing tests were those who later experienced extubation failure. The reintubation rate was 24.1%, and the main determinant of reintubation (in half of these patients) was dysphagia, followed by respiratory complications [24]. Prolonged endotracheal intubation is a major cause of swallowing dysfunction, and the determination of extubation readiness is challenging in this population. The criteria used in the general population for the prediction of extubation failure are based on airway parameters and lung mechanics, whereas in stroke patients, neurological status has a prominent role in the development of dysphagia and is a leading contributor to poor outcome [25]. The prevalence of post-extubation dysphagia ranges from 12 to 69% in the general ICU population, reaching 93% in neurological patients [24]. The main causes of post-extubation dysphagia are cerebrovascular disease due to damage to the central swallowing network, pharyngo-laryngeal lesions, neuropathies, and myopathies, as well as sedation and mucosal damage [24]. Although several techniques have been developed to diagnose dysphagia, only videofluoroscopy has been considered the goal standard for swallowing dysfunction. An interventional study using fiberoptic endoscopy and including general ICU patients within 48 h from extubation found that post-extubation dysphagia could be diagnosed in 50% of patients, and the overall incidence of aspiration events was 56%, of which 25% were silent [25]. This technique demonstrated its utility in the diagnosis of post-extubation dysphagia in 69.3% of patients [26]. In summary, current evidence agrees on the utility and feasibility of bedside fiberoptic endoscopic swallowing study for assessment of post-extubation dysphagia in patients at risk for aspiration and swallowing dysfunction.

## Ventilator settings

Due to the high incidence of respiratory complications in stroke patients, optimal ventilator management is mandatory [5]. The main goal of MV should be to maintain appropriate oxygen levels and tight control of carbon dioxide tension (PaCO_2_), without inducing ventilator-associated lung damage.

### Oxygen therapy

Hypoxia is common in stroke patients, and an oxygen saturation (SaO_2_) below 90% in the first few hours after hospital admission is associated with a twofold risk of mortality [27]. Supplemental oxygen could prevent hypoxia and additional neurological deterioration, but it could also lead to adverse effects; in fact, hyperoxia is independently associated with mortality in brain-injured patients, with a higher risk of delayed cerebral ischaemia and poor outcome [28]. Oxygen may increase the risk of respiratory tract infections due to contamination, has a direct effect on vascular tone and blood pressure [29], and at high concentrations, causes vasoconstriction and pulmonary toxicity due to the formation of toxic free radicals. Oxidative stress is also responsible for the activation of cell signalling pathways, inducing apoptosis and neuronal cell death [28, 29]. Evidence from well-designed trials of oxygen supplementation after acute stroke is conflicting and insufficient to guide clinical practice (Table 1). A quasi-randomised study of oxygen supplementation for acute stroke has shown that routine oxygen therapy in stroke patients does not reduce morbidity and mortality [30]. The Stroke Oxygen Pilot Study (SO_2_S), a multicentre randomised controlled trial of oxygen therapy during the first 3 days in patients with acute stroke, compared continuous low-dose oxygen therapy with a nasal cannula at 2–3 L/min versus nocturnal low-dose versus usual care (oxygen only when needed) [31]. The authors observed that routine prophylactic low-dose oxygen supplementation did not improve outcome at 3 months [32]. In patients with stroke who have a low arterial oxygen level, adjunctive oxygen treatment is warranted, whereas in non-hypoxic patients, preventive oxygen therapy is not recommended [32] (Table 2). Recent guidelines on the management of traumatic brain injury recommend to set partial pressure of oxygen (PaO_2_) also considering brain partial tension of oxygen (PbtO_2_) or jugular venous saturation (SjvO_2_) values and to maintain normoxia [33]. For stroke patients, the same recommendations could be acceptable, but specific studies are required. In addition, stroke patients are particularly susceptible to sleep-disordered breathing, with a prevalence of obstructive sleep apnoea ranging from 30 to 80%. A meta-analysis of 29 studies showed that sleep-disordered breathing is present in up to 72% of stroke patients, whether haemorrhagic or ischaemic, without significant differences between stroke subpopulations. Only 7% of patients suffered from central sleep apnoea, thus suggesting that young stroke patients are more susceptible to obstructive sleep apnoea than their age-matched peers who have not had a stroke [34]. Therefore, the use of polysomnography, limited channel devices, and auto-continuous positive airway pressure should be implemented for the management of sleep apnoea in patients after a stroke or transient ischaemic attack [34].

**Table 1:** Studies evaluating the use of oxygen therapy in acute ischaemic stroke patients

Authors	Design	Aim	Participants	Total (*n*)	O_2_ therapy	Conclusions
Rønning et al. 1999 [30]	Quasi-randomised trial	To test the hypothesis that breathing 100% O_2_ for the first 24 h after an AS would not reduce mortality, impairment, or disability	AS	555 (66/555 ICH)	O_2_ therapy (100% atm, 3 L/min for 24 h [*n* = 292]) versus controls (no additional O_2_)	Supplemental O_2_ should not routinely be given to non-hypoxic stroke victims with minor or moderate strokes.
Ali et al. 2005 [35]	Prospective study	To assess the effects of different doses and routes of O_2_ administration on SatO_2_ in patients with stroke	AS	21 (15 AIS; 6 ICH)	Steps: -Room air for 30 min; − 2-3 L/min of O_2_ (nasal cannula) -FiO_2_ 0.24 (VM) -FiO_2_ 0.3535% (VM) -Room air	There was a dose-response relationship between the amount of O_2_ given and the resultant changes in SatO_2_.
Singhal et al. 2005 [36]	Randomised trial	To investigate the effects of high-flow oxygen (HFO) in AIS	AIS	16	HFO (humidified O2 at 45 L/min via facemask for 8 h [*n* = 9]) versus controls (room air or nasal O_2_ 1–3 L/min to maintain SaO_2_ 95% [*n* = 7])	HFO is associated with a transient improvement of clinical deficits and MRI abnormalities in select patients with AIS.
Chiu et al. 2006 [37]	Prospective study	To investigate the feasibility of eubaric hyperoxia therapy by VM in a group of patients who experienced a severe AIS	AIS	46	O_2_ therapy (FiO_2_ 0.4via VM [*n* = 17]) versus controls (nasal cannula [*n* = 29])	By using VM therapy with a FiO_2_ of 0.4, there might be less mortality and comorbidities in treated patients who experienced a severe AIS.
Singhal et al. 2007 [38]	RCT	To investigate the metabolic effects of normobaric oxygen (NBO) on AIS brain tissues using MRSI and diffusion-perfusion MRI	AIS	6	NBO (45 L/min O_2_ via face mask for 8 h [*n* = 4]) versus controls (room air [*n* = 2])	NBO improves aerobic metabolism and preserves neuronal integrity in AIS brain.
Padma et al. 2010 [39]	Randomised trial	To study the role of NBO in AIS in Indian patients	AIS	40	NBO (10 L/min O_2_ for 12 h [*n* = 20]) versus controls (room air/2 L/min O_2_ via face mask to maintain SaO2 ≥ 95% [*n* = 20])	NBO did not improve the clinical scores of stroke outcome in Indian patients with AIS.
Roffe et al. 2011 [31]	RCT	To report the effects of routine use of O_2_ supplementation for 72 h on SatO_2_ and neurological outcomes at 1 week after an AS	AS (clinical diagnosis)	289 (257 AIS, 24 ICH, 8 undetermined)	O_2_ therapy (O_2_ via nasal cannula for 72 h [*n* = 148]) or controls (room air/O_2_ if clinically indicated [*n* = 141])	Routine O_2_ supplementation started within 24 h of hospital admission with AS led to a small, but statistically significant, improvement in neurological recovery at 1 week. The difference in NIHSS improvement may be due to baseline imbalance in stroke severity between the two groups.
Ali et al. 2013 [40]	RCT	To report the effects of routine O_2_ supplementation for 72 h on SatO_2_ and neurological outcomes at 6 months after AS	AS (clinical diagnosis)	289 (257 AIS, 24 ICH, 8 undetermined)	O_2_ therapy (O_2_ via nasal cannula for 72 h [*n* = 148]) or controls (room air/O_2_ if clinically indicated [*n* = 141])	None of the key outcomes differed at 6 months between the groups. Although not statistically significant and generally of small magnitude, the effects were predominantly in favour of the O_2_ group.
Jeon et al., 2014 [28]	Prospective, observational cohort study	To determine the association between exposure to hyperoxia and the risk of DCI after SAH	SAH	252	Hyperoxia (the highest quartile of an area under the curve of PaO_2_, until the development of DCI [PaO_2_ ≥ 173 mmHg])	Exposure to excess O_2_ after SAH may represent a modifiable factor for morbidity and mortality in this population.
Rincon et al. 2014 [41]	Retrospective multicentre cohort study	To test the hypothesis that hyperoxia was associated with higher in-hospital mortality in ventilated AS patients admitted to the ICU	AS	2894 (554 AIS, 936 SAH, 1404 ICH)	O_2_ therapy to obtain PaO_2_ ≥ 300 mmHg	Hyperoxia was an independent predictor of in-hospital death.
Mazdeh et al. 2015 [42]	RCT	To evaluate the effects of normobaric hyperoxia on clinical outcomes of patients with severe AS	AS	52	O_2_ therapy (VM for 12 h) versus controls (no O_2_)	NBO therapy in the first 12 h of AS could improve long-time outcome of the patients with either ischaemic or haemorrhagic stroke.
Roffe et al., 2017 [32]	Multicentre single-blind RCT	To assess whether routine prophylactic use of low-dose O_2_ therapy was more effective than control O_2_ administration at reducing death and disability at 90 days, and if so, whether O_2_ given at night only, when hypoxia is most frequent, and O_2_ administration is least likely to interfere with rehabilitation, was more effective than continuous supplementation	AS (clinical diagnosis)	8003 (6555 AIS, 588 ICH, 294 AS without CT diagnosis, 168 TIA, 292 non-stroke diagnoses, 106 missing data)	Continuous O_2_ (2–3 L/min via nasal cannula for 72 h [*n* = 2668]) versus nocturnal O_2_ (2–3 L/min via nasal cannula for 3 nights [*n* = 2667]) versus controls (O_2_ if clinically indicated [*n* = 2668])	Among non-hypoxic patients with AS, the prophylactic use of low-dose O_2_ supplementation did not reduce death or disability at 3 months.
Ding et al. 2018 [43]	Meta-analysis	To analyse the current data of NBO on brain protection as used in the clinical settings	AS	6366	NBO group (… [*n* = 3207]) versus controls [*n* = 3159]	The existing trends toward benefits revealed in this meta-analysis help us appreciate the promising value of NBO, although current evidence of NBO on improving clinical outcomes of stroke is insufficient.
Roffe et al. 2018 [44]	Multicentre, prospective, randomised, open, blinded-end point trial	(1) To assess whether or not routine low-dose of O_2_ supplementation in patients with AS improves outcome compared with no O_2_ and (2) to assess whether or not O_2_ given at night only, when SatO_2_ is most likely to be low, is more effective than continuous supplementation	AS (clinical diagnosis)	8003 (6555 AIS, 588 ICH, 168 TIA, 292 non-stroke diagnoses, 106 missing data)	Continuous O_2_ (2–3 L/min via nasal cannula for 72 h [*n* = 668]) versus nocturnal O_2_ (2–3 L/min via nasal cannula for 3 nights [*n* = 2667]) versus controls (O_2_ if clinically indicated [*n* = 2668])	Routine use of low-dose O_2_ supplementation in stroke patients who are not severely hypoxic is safe but does not improve outcome after AS.

*AIS* acute ischaemic stroke, *AS* acute stroke (including haemorrhagic), *atm* atmospheres, *DCI* delayed cerebral ischaemia, *FiO*_*2*_ fraction of inspired oxygen, *HFO* high-flow oxygen, *ICH* intracerebral haemorrhage, *MRI* magnetic resonance imaging, *MRSI* multivoxel magnetic resonance spectroscopic imaging, *NBO* normobaric oxygen, *PaO*_*2*_ partial pressure of oxygen, *RCT* randomised controlled trial, *SatO*_*2*_ oxygen saturation, *SDH* subdural haemorrhage, *TIA* transient ischaemic attack, *VM* venturi mask

**Table 2:** Respiratory management of patients with stroke according to 2018 AHA/ASA guidelines [23]

Intubation and ventilation are recommended in patients with decreased consciousness, bulbar dysfunction with inability to protect the airway, or intracranial hypertension (level I).	
Aim for normoxia and normocapnia (NA).	
Continuous monitoring of oxygenation is strongly recommended in patients with AIS in ICU. Supplemental oxygen should be administered if SpO_2_ > 94% (level I).	
Supplemental oxygen is not recommended in non-hypoxic patients (level III).	
Hyperbaric oxygen is not recommended, except in case of air embolism (level III).	

*AIS* acute ischemic stroke, *ICU* intensive care unit, *SpO2* oxygen saturation

### Invasive ventilation

#### Intubation

There are several commonly accepted indications for intubation of neurological patients (Table 3) [45]. In general, the decision to intubate is often triggered by neurological deficits, such as a Glasgow Coma Score (GCS) < 9, signs of increased intracranial pressure, generalised (tonic-clonic) seizures, infarct size > 2/3 of the middle cerebral artery territory, and midline shift on imaging [23]. Patients with acute brain injury, including stroke, may be at risk for difficult intubation, especially if there is suspicion of associated trauma. Predicting a difficult airway is crucial for the selection of appropriate techniques (i.e. awake fiberoptic versus rapid sequence induction) and tools (video versus direct laryngoscopy) [46, 47]. In non-emergency settings, awake intubation is the procedure of choice in case of anticipated difficult airway or ventilation, except for patients with elevated intracranial pressure (ICP). In this case, rapid sequence intubation is preferred, since it limits the elevation of ICP induced by laryngoscopy [45]. Adequate oxygenation during intubation is essential for patients with stroke, to prevent secondary injury of the vulnerable brain and further ICP elevation [45–47]. In critically ill patients, 3 min of pre-oxygenation with non-invasive positive pressure ventilation [48] or a heated high-flow nasal cannula (HHFNC) at 60–70 L/min [49] may be more effective than pre-oxygenation with a high-flow (non-rebreather) face mask. However, some evidence suggests the use of apnoeic oxygenation (with HHFNC at 60–70 L/min or a nasal cannula at 15 L/min) during laryngoscopy [45, 49]. Indeed, HHFNC was associated with less desaturation in more hypoxaemic patients when compared to positive pressure ventilation [50]. Since pre-oxygenation with 100% oxygen is easy to perform and has no serious adverse effects, its use is recommended during intubation of stroke patients. Once intubation is achieved, oxygen concentration should be decreased immediately to the lowest FiO_2_ that will result in a SatO2 ≥ 95% [50].

**Table 3:** Causes of intubation in stroke patients

GCS < 9	
Airway compromise	
Apnoea	
Hypoxaemia despite supplemental oxygen	
Impaired swallowing and gag reflexes	
Inability to clear secretions	
Seizures or drugs suppressing respiratory drive	
Need for intracranial pressure management	
Anticipated neurological or cardiopulmonary decline requiring transport or immediate treatment	

*GCS* Glasgow Coma Scale

### Ventilator settings

Hypoxaemia and hyper/hypocapnia should be avoided to decrease the risk of secondary brain injury. PaCO_2_ is a powerful determinant of cerebral blood flow [33]; however, not only hypercapnia is dangerous in stroke patients, but also hyperventilation, because cerebral vasoconstriction can cause brain tissue hypoxia and compromise cerebral compliance and blood flow [51]. No consensus is available regarding the optimal respiratory rate and tidal volume (*V*_T_) to achieve the PaCO_2_ target, with higher *V*_T_ being generally applied in this group of patients [11]. However, after severe brain injury, higher *V*_T_ is associated with both an increased risk of acute lung injury and acute intracranial hypertension [9, 52]. Similarly, higher driving pressure is associated with a higher risk for ARDS [52]. Moreover, in patients recovered from acute lung injury, the shorter is the inspiratory rise time, the shorter will be the inspiratory work of breathing; also, at 15 cmH_2_O of pressure support ventilation, the lowest cycling off criteria reduced respiratory rate and increased *V*_T_ [53]. In critically ill patients requiring MV, protective ventilation with a *V*_T_ of 6 mL/kg, adequate positive end-expiration pressure (PEEP) levels and limiting plateau pressure to < 30 cmH_2_O has shown to reduce absolute mortality by 10% [54]. However, clinical trials testing ventilation strategies designed for lung protection frequently excluded brain-injured patients; therefore, ventilator management has been scarcely evaluated in neurocritical settings. Indeed, in critically ill patients with acute brain injury, the use of protective lung ventilation is controversial and might even be contraindicated, as it can increase ICP due to permissive hypercapnia and the use of high airway pressures during recruitment manoeuvres [55]. In a porcine model of acute lung injury, major improvement in cerebral oxygenation was observed in animals ventilated with low *V*_T_ when compared with high *V*_T_ [56]. A multicentre trial with 749 brain-injured patients (36% in the pre-intervention group and 46% in the intervention group were stroke patients) [57] suggested that protective ventilation (*V*_T_ ≤ 7 mL/kg of ideal PBW and PEEP 6–8 cmH_2_O) can significantly improve the number of ventilator-free days at day 90, as well as reduce the mortality rate. No specific studies focusing on optimal ventilator settings in patients with stroke are available, and only a minority of patients included in the large trials exploring the role of protective ventilation on outcome had stroke; we recommend keeping Pplat between 18 and 25 cmH_2_O and minimising respiratory rate to reduce mechanical power. Future studies are needed to better define the optimal *V*_T_ in stroke patients requiring MV.

### PEEP

Application of PEEP improves arterial oxygenation. However, in brain-injured patients, caution is warranted because PEEP may worsen or trigger intracranial hypertension [51, 58]. Mascia et al. suggested that ICP increases significantly when PEEP is applied, but only in patients where PEEP induces alveolar hyperinflation with a consequent increase in PaCO_2_, whereas ICP remains constant when PEEP causes alveolar recruitment with no change in PaCO_2_ [58]. In an experimental study in both healthy and stroke pigs, high PEEP levels (up to 25 cmH_2_O) did not impair ICP, cerebral oxygenation, or regional cerebral blood flow [59]. Application of high PEEP (up to 20 cmH_2_0) in patients with haemorrhagic stroke resulted in a significant decrease in mean arterial pressure (MAP) and regional cerebral blood flow, but further analyses showed that changes in regional cerebral blood flow depend on MAP changes as a result of impaired cerebrovascular autoregulation [59]. Georgiadis et al. showed that PEEP can be safe in patients with acute stroke, even after increasing PEEP levels up to 12 mmHg, providing that MAP is maintained [60]. In addition, a recent trial in non-ARDS patients demonstrated the non-inferiority of a low-*V*_T_, low-PEEP strategy as compared to high *V*_T_ [61]. Therefore, PEEP is considered safe if (1) haemodynamic status and euvolaemia are maintained to minimise the effects of PEEP on cerebral perfusion pressure and (2) the value of PEEP is lower than ICP, to avoid a decrease in venous outflow [55], thus resulting in a beneficial effect on brain oxygenation [62] while (3) avoiding excessive over-distention and keeping the lung at rest [63].

### Recruitment manoeuvres

Recruitment manoeuvres (RMs) may improve pulmonary gas exchange and respiratory mechanics [64]. However, they may also cause intracranial hypertension by impairing jugular venous outflow and impeding cerebral venous return to the right atrium [64]. In a study by Bein and colleagues of patients with brain injury (6 of 11 were stroke patients) and acute lung injury, volume RMs (which included a 30-s progressive increase in peak pressure up to 60 cmH_2_O and a sustained pressure at the same level for the next 30 s) led to a deterioration of cerebral oxygenation and, simultaneously, a reduction of MAP and an increase in ICP, with critical reduction of the cerebral perfusion pressure. In 9 brain-injured patients (of whom 6 were stroke patients), a stepwise RM with 3-cmH_2_O intermittent increments and decrements of PEEP was applied [65], resulting in a positive correlation between PEEP and ICP and a negative correlation between PEEP and cerebral perfusion pressure (CPP). These data suggest that the effects of PEEP on blood pressure and cerebral perfusion pressure vary greatly in acutely brain-injured patients and that strict MAP and ICP monitoring is of benefit when PEEP is applied. Therefore, RMs could be safely performed when indicated in patients with both stroke and lung injury, especially if setting a PEEP level lower than the ICP and maintaining an effective MAP [66].

### Prone positioning

Similarly to RMs, prone positioning has demonstrated to be an efficient technique to improve oxygenation but should be used cautiously in patients with stroke and reduced intracranial compliance, as it can increase intrathoracic pressure and raise ICP [67]. Only one study investigating the effects of the prone position on respiratory failure provided data on cerebral haemodynamic in patients with stroke, with contradictory results [68]. Thus, the benefits and risks of prone positioning in patients with stroke are still unclear. Nekludov et al. found a significant improvement of pulmonary oxygenation, but a rise in ICP in a group of 8 patients with a GCS ≤8 (3 of them with stroke) and pulmonary injury [67]. Thelandersson and colleagues investigated 11 neurological patients (5 of them with stroke) with FiO_2_ > 0.4 in prone position for only 3 h, and also found an increase in systemic oxygenation, but unlike the previous study, without significant changes in ICP or MAP [69]. Altogether, prone positioning can present challenges in stroke patients because of the risk of developing intracranial hypertension and displacement of neuromonitoring tools. However, it could be taken into consideration in patients with severe, refractory hypoxaemia and under strict multimodal neuromonitoring.

### Other ventilation strategies

There are insufficient studies to evaluate the role and safety of high-frequency oscillatory ventilation (HFOV) in stroke population. Theoretically, the application of higher mean airway pressures could decrease cerebral venous drainage, and the PCO_2_ clearance could be insufficient; both aspects are unadvisable in severe brain-injured patients. Very few observational studies have evaluated the use of HFOV in adults with acute brain injury, with even fewer focusing on stroke [70]. In a systematic review of the effects of HFOV on cerebral perfusion pressure and intracranial pressure, the authors concluded that, for patients with traumatic brain injury and ARDS, HFOV may improve oxygenation and increase PaCO_2_. Thus, if HFOV is considered, continuous monitoring of oxygen saturation, MAP, PaCO_2_, and ICP is paramount [70].

In case of life-threatening hypoxia refractory to conventional MV, the use of rescue therapies has been encouraged to re-establish adequate levels of oxygenation while minimising ventilator-associated injury. In selected cases, extracorporeal lung support techniques have been considered [71].

In summary, in stroke patients requiring MV, protective ventilation with close monitoring of neurological and respiratory variables is essential to ensure lung safety and avoid secondary brain injury. Furthermore, monitoring of cerebral oxygenation should be used when possible to allow the clinician to discriminate between normal and critically impaired tissue oxygenation and help refine ventilator settings [9, 55]. In future, non-invasive cerebral monitoring could be implemented to individualise ventilator settings [72].

#### Extubation

Neurological patients experienced prolonged ICU stays and mechanical ventilation, a higher rate of early tracheostomy, and higher incidence of ventilator-associated pneumonia compared to non-neurological patients [5]. Although often necessary and life-saving, MV is time-dependently associated with various complications and may increase morbidity and mortality [73]. Therefore, weaning from mechanical ventilation has been suggested to take place as early as is safely possible. Moreover, there is evidence that neurological patients are especially prone to extubation failure, with high reintubation rates, ranging from 20 to 40% [74]. Prediction of successful extubation is critical, as both delayed and premature extubation increase complication rates need for tracheostomy, duration of ICU stay, and mortality [75]. Unfortunately, classic respiratory predictors for successful extubation in general critical care (such as *V*_T_, respiratory rate, negative inspiratory force, forced vital capacity, rapid shallow breathing index, and PaO_2_/FiO_2_) are unreliable in brain-injured patients [76]. The level of consciousness at the moment of extubation could be considered a risk factor for extubation failure [77], but a recent study [73] showed controversial results. In a meta-analysis [74] specifically looking for predictors of extubation failure in neurocritical care patients, a low GCS (7–9) was identified as a risk factor, with a nearly fivefold increased risk of reintubation. Other factors included the ability to follow commands, secretion texture, and presence of a gag reflex. In a small retrospective study of middle cerebral artery stroke patients, a composite GCS score of 8 with an eye subscore of 4 was associated with successful extubation [78]. However, GCS alone cannot be considered a predictive factor for extubation success, as it has never been validated in intubated patients whose verbal component is impossible to assess and often arbitrarily scored [79]. More recently, Godet et al. [73] developed a clinical score to predict extubation failure in the general brain-injured population with a GCS < 12 before tracheal intubation, intubated for neurological reasons, and ventilated for more than 48 h; it includes upper airway functions (e.g. gag reflex, cough, and deglutition) and neurological status (evaluated through the visual subscale of the Coma Recovery Scale-Revised). However, external validation of this score is required, and no data specific to stroke patients are available. Recent data have evaluated new physical features in patients with severe brain injury. In a multicentre study, age < 40 years, visual pursuit, attempts at swallowing, and a GCS > 10 were found to be predictors of successful extubation [80]. On the basis of these items, the VISAGE score was constructed. If three or more items are positive, an extubation success rate of 90% can be expected. Visual pursuit and preserved upper airway reflexes [73], younger age [81], negative fluid balance, and the presence of cough have been described as favourable signs of extubation success by other observational studies. Further studies are warranted to confirm the findings of the latest studies on neurological patients in general and stroke in particular.

### Tracheostomy

Up to 45% of patients admitted to the ICU for stroke require tracheostomy (TT) [81] versus 10–15% in the general ICU population [5]. Recognised advantages of tracheostomy compared with prolonged oro-tracheal intubation are a reduction of pharyngeal and laryngeal lesions, a better oral hygiene and nursing care, and higher patient comfort [4]. Predictors that may suggest the decision to perform a tracheostomy in stroke patients have been described in few studies [82, 83]. Different types of stroke are associated with the need for tracheostomy, including large hemispheric stroke, basilar thrombosis, brainstem infarction, and space-occupying cerebellar stroke [82]. Indeed, the location and extension of brain damage and its secondary effects (including cerebral oedema and secondary ischaemia) may influence the need for MV support. In particular, the compromise of the brain regions which regulate breathing (respiratory centres in the pons, or medulla), level of consciousness (reticular formation, thalami), and swallowing (medulla and brainstem) may lead to respiratory failure and need for tracheostomy [82]. There are two main clinical scenarios in which mechanical ventilation and tracheostomy are usually considered after stroke. The first is in patients with such an extensive stroke that ICU admission and MV are required. In these, long-term ventilation and prolonged insufficient airway protection if extubation fails or is deemed infeasible are the main indications for tracheostomy [82, 83]. The second scenario is in patients with stroke of only moderate overall severity, but which affects the swallowing centres of the brain (such as infarcts of the brainstem or the medulla oblongata), causing dysphagia. In this case, the decision to perform a tracheostomy can be related to severe dysphagia posing a risk of aspiration [84].

In a retrospective study by Szeder et al. [85] on patients with intracranial haemorrhage, features associated with the need for TT were GCS < 9 at day 3 as well as radiological findings such as hydrocephalus, midline shift, and intraventricular haemorrhage. Schonenberger et al. [83] proposed the stroke-related early tracheotomy (SET) score to screen patients for prolonged intubation and need for TT by combining neurological parameters (e.g. dysphagia and GCS < 10), neuroimaging features (e.g. brainstem lesion, hydrocephalus, intracranial haemorrhage volume > 25 mL), general organ function, and surgical procedures. Furthermore, Steidl et al. conducted a two-centre, prospective observational study on both ischaemic and haemorrhagic stroke patients to evaluate the extubation rate over tracheostomy. Overall, 47% of patients were tracheostomised without any extubation attempt, whereas 53% were primarily extubated. Among patients primarily tracheotomised, most had haemorrhagic stroke, higher National Institute of Health Stroke Scale, and lower Glasgow Coma scores on admission. Additionally, they received more neurosurgical treatments as compared to those extubated [86]. As can be deduced by the literature, the optimal timing of TT in stroke patients is still unclear. Potential benefits of early TT (such as ventilation duration and length of stay, ventilator weaning, airway safety, rate of pneumonia, outcome, and mortality) have yet to be proven [4]. McCann et al. demonstrated a reduced length of stay in patients undergoing earlier (within 1 week) tracheostomy and percutaneous endoscopic gastrostomy (PEG) placement in patients with haemorrhagic stroke [87]. A recent meta-analysis by McCredie et al. evaluated the effect of early versus late TT or prolonged endotracheal intubation in acute brain-injured patients. Ten trials met the selection criteria. Early TT reduced long-term mortality and duration of mechanical ventilation, but insufficient trials were available to perform subgroups analysis; thus, the study refers to the general neuro-ICU population, and no specific data are reported about stroke patients [88]. Overall, as procedural risk is low and early tracheostomy does not seem to affect the clinical course of the ventilated stroke patient, it is suggested that ventilation needs be assessed after the first week of intensive care and tracheostomy considered if an extubation attempt failed or is judged not to be feasible [4].

## Recommendations and conclusions

**Fig. 3:**
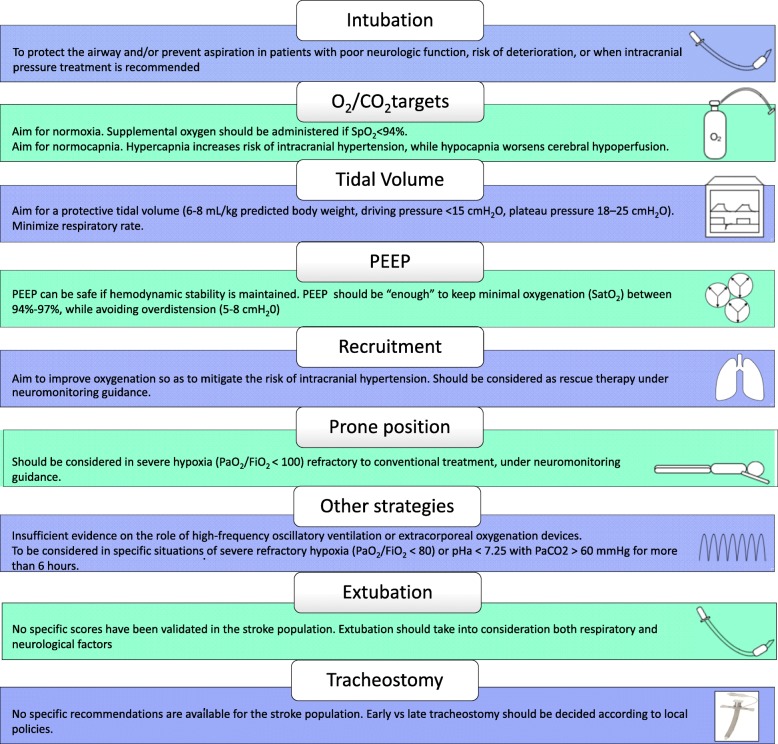
Recommended mechanical ventilation strategies for patients with acute ischaemic stroke. Abbreviations: PEEP, positive end-expiratory pressure; O_2_, oxygen; CO_2_, carbon dioxide

Although there are no specific data regarding the effect of respiratory management on stroke patients’ outcomes, specific ventilator strategies in this population could potentially improve neurologic outcome and prevent respiratory failure by correcting modifiable risk factors or controlling brain-induced inflammation of distal organs, mainly the lungs. Protective ventilation has to be considered in this population to obtain the target of normoxia and normocapnia avoiding high *V*_T_. Because of the lack of clear evidence and the poor quality of the studies available, clinical decisions regarding ventilator strategies in stroke patients should be made on a case-by-case basis, considering patients’ characteristics, risks, and benefits, as well as prognosis and neurological status, using multimodal brain monitoring to assess cerebral haemodynamic and oxygenation and to evaluate the effects of protective ventilation (in particular of PaCO_2_ and PEEP). We therefore propose a list of recommendations based on the available literature (Fig. 3). Further trials are needed to guide ventilation management in acute ischaemic stroke and to assess possible disparities between the different aetiologies of stroke.

## Data Availability

NA

## Abbreviations

ARDS: Acute respiratory distress syndrome
CBF: Cerebral blood flow
ICU: Intensive care unit
AIS: Acute ischaemic stroke
SAH: Subarachnoid haemorrhage
ICH: Intracranial haemorrhage
IVH: Intraventricular haemorrhage
TBI: Traumatic brain injury
MV: Mechanical ventilation
SAP: Stroke-associated pneumonia
PBW: Predicted body weight
*V*_T_: Tidal volume
BBB: Blood-brain barrier
MAP: Mean arterial pressure
CNS: Central nervous system
LPS: Lipopolysaccharide
IL-6: Interleukin-6
PEEP: Positive end-expiratory pressure
VAP: Ventilator-associated pneumonia
GCS: Glasgow Coma Scale
PaO_2_: Partial pressure of arterial oxygen
ONSD: Optic nerve sheath diameter
ICP_PI_: Pulsatility index
ICP_FVd_: Diastolic velocity
TCD: Transcranial Doppler
PaCO_2_: Partial pressure of carbon dioxide
PbtO_2_: Brain tissue oxygen tension
TT: Tracheostomy
PDT: Percutaneous dilatational tracheostomy
ST: Open tracheostomy
EtCO_2_: End-tidal CO_2_
SjvO_2_: Jugular venous saturation
ESO: European Stroke Organisation
ASA: American Stroke Association
NICE: National Institute for Health and Care Excellence
CMRO_2_: Cerebral metabolic rate for oxygen
ICP: Intracranial pressure
NIV: Non-invasive ventilation
HHFNC: Heated high-flow nasal cannula
PEEP: Positive end-expiration pressure
SaO_2_: Oxygen saturation
CPP: Cerebral perfusion pressure
Crs: Respiratory system compliance
FiO_2_: Inspired oxygen fraction
RM: Recruitment manoeuvres
